# Recombinant Sox Enzymes from *Paracoccus pantotrophus* Degrade Hydrogen Sulfide, a Major Component of Oral Malodor

**DOI:** 10.1264/jsme2.ME16140

**Published:** 2017-03-03

**Authors:** Atik Ramadhani, Miki Kawada-Matsuo, Hitoshi Komatsuzawa, Takahiko Oho

**Affiliations:** 1 Department of Preventive Dentistry, Kagoshima University Graduate School of Medical and Dental Sciences8–35–1 Sakuragaoka, Kagoshima 890–8544Japan; 2 Department of Oral Microbiology, Kagoshima University Graduate School of Medical and Dental Sciences8–35–1 Sakuragaoka, Kagoshima 890–8544Japan

**Keywords:** hydrogen sulfide, oral bacteria, oral malodor, *Paracoccus pantotrophus*, sulfur-oxidizing enzyme

## Abstract

Hydrogen sulfide (H_2_S) is emitted from industrial activities, and several chemotrophs possessing Sox enzymes are used for its removal. Oral malodor is a common issue in the dental field and major malodorous components are volatile sulfur compounds (VSCs), including H_2_S and methyl mercaptan. *Paracoccus pantotrophus* is an aerobic, neutrophilic facultatively autotrophic bacterium that possesses sulfur-oxidizing (Sox) enzymes in order to use sulfur compounds as an energy source. In the present study, we cloned the Sox enzymes of *P. pantotrophus* GB17 and evaluated their VSC-degrading activities for the prevention of oral malodor. Six genes, *soxX*, *soxY*, *soxZ*, *soxA*, *soxB*, and *soxCD*, were amplified from *P. pantotrophus* GB17. Each fragment was cloned into a vector for the expression of 6×His-tagged fusion proteins in *Escherichia coli*. Recombinant Sox (rSox) proteins were purified from whole-cell extracts of *E. coli* using nickel affinity chromatography. The enzyme mixture was investigated for the degradation of VSCs using gas chromatography. Each of the rSox enzymes was purified to apparent homogeneity, as confirmed by SDS-PAGE. The rSox enzyme mixture degraded H_2_S in dose- and time-dependent manners. All rSox enzymes were necessary for degrading H_2_S. The H_2_S-degrading activities of rSox enzymes were stable at 25–80°C, and the optimum pH was 7.0. The amount of H_2_S produced by periodontopathic bacteria or oral bacteria collected from human subjects decreased after an incubation with rSox enzymes. These results suggest that the combination of rSox enzymes from *P. pantotrophus* GB17 is useful for the prevention of oral malodor.

Hydrogen sulfide (H_2_S) is emitted from industrial activities, particularly from biogas and protein-rich industrial waste. H_2_S exhibits high toxicity and is very corrosive to internal combustion engines. Many commercial chemical technologies are currently used to remove H_2_S in industry (41). However, these chemical H_2_S removal processes are expensive due to high chemical requirements as well as energy and disposal costs, and mostly exert short-term effects only. Biological treatment methods for the removal of H_2_S are desirable, and several studies have investigated this issue (10, 32). The oxidation of H_2_S is mediated by various aerobic lithotrophic and anaerobic phototrophic bacteria (35).

In the dental field, oral malodor, the presence of unpleasant or foul-smelling breath, is a common issue worldwide and may have significant social or psychological effects on those affected by it (6, 15, 21). Oral malodor is a mixture of malodorous components, including volatile sulfur compounds (VSCs), such as H_2_S, methyl mercaptan (CH_3_SH), and dimethyl sulfide (6, 15). VSCs are produced in saliva, gingival crevices, on the tongue surface, and in other areas via the putrefactive activities of microorganisms on sulfur-containing amino acids, such as cysteine and methionine (3, 6). H_2_S and CH_3_SH are primarily responsible for oral malodor and comprise approximately 90% of the VSC content in mouth air (43).

VSCs also exert adverse effects on oral tissues. Previous studies demonstrated that H_2_S damages gingival epithelial cells (42), increases the permeability of the oral mucosa *in vitro* (24), and causes apoptosis in human gingival fibroblasts (2). H_2_S has predominantly been detected in pockets (27) associated with periodontal bacteria, including *Fusobacterium nucleatum*, one of the most active oral bacteria to produce H_2_S from L-cysteine (26). The presence of CH_3_SH is also known to be involved in the induction or progression of periodontal disease. Exposure to CH_3_SH inhibits cell migration in periodontal ligament cells (18) as well as epithelial cell growth and proliferation (38). *Porphyromonas gingivalis*, a black-pigmented anaerobic bacterium and major pathogen in adult periodontitis, produces large amounts of CH_3_SH in human serum (26) from L-methionine.

Several attempts have been made to prevent and reduce oral malodor (6, 40, 45). Mechanical prophylaxis using a toothbrush and tongue scraper represents a basic method to remove bacterial cells and substrates from the oral cavity; however, organisms still grow and accumulate. Mouth rinses containing antimicrobial agents, such as chlorhexidine and cetylpyridinium chloride, have been used to decrease bacterial numbers, leading to a reduction in oral malodor (9, 47). Additionally, zinc ions have been used to reduce VSCs through their oxidizing effects on the thiol groups in VSC precursors (1, 16). However, the effects of specifically formulated mouth rinses on oral malodor generally remain unclear (40). Therefore, new methods to control VSCs in the oral cavity need to be established for the prevention of oral malodor.

The purpose of this study was to examine the applicability of the sulfur-oxidizing (Sox) enzyme system to the control of oral malodor. We selected the sulfur-oxidizing bacterium, *Paracoccus pantotrophus*, which is an aerobic, Gram-negative, neutrophilic facultatively autotrophic bacterium that grows using thiosulfate or molecular hydrogen as an energy source and heterotrophically using various carbon sources (13). The Sox enzyme system in *P. pantotrophus* has already been examined. The *sox* gene region of *P. pantotrophus* comprises 12 open reading frames and seven genes, soxXYZABCD, which encode proteins that are essential for sulfur oxidation *in vitro* (34). The Sox proteins of *P. pantotrophus* are located in the periplasm (12), and four proteins, SoxXA, SoxYZ, SoxB, and SoxCD, are known to be required for the H_2_S-, sulfur-, thiosulfate-, and sulfite-dependent reduction of horse cytochrome *c* (34). We report the cloning of *sox* genes from *P. pantotrophus* GB17—*soxX*, *soxY*, *soxZ*, *soxA*, *soxB*, and *soxCD*—and the characteristics of recombinant Sox (rSox) enzymes. We investigated the rSox enzymatic degradation of VSCs produced by periodontopathic bacteria and oral bacteria.

## Materials and Methods

### Bacterial strains and culture conditions

All strains were cultivated at 37°C. *P. pantotrophus* GB17 (NBRC 102493) was obtained from NBRC (Kisarazu, Japan) and used throughout this study. Seed cultures were grown aerobically in brain heart infusion (BHI; Becton, Dickinson and Company, Sparks, MD, USA) broth supplemented with 4 mM magnesium sulfate. *Escherichia coli* XL II was grown aerobically in Luria Bertani (LB; Difco Laboratories, Detroit, MI, USA) medium. *P. gingivalis* W83 was grown anaerobically in GAM broth (Nissui Medical, Tokyo, Japan) supplemented with hemin (5 μg mL^−1^) and menadione (1 μg mL^−1^). *F. nucleatum* ATCC10953 was grown anaerobically in BHI broth supplemented with 5 mg mL^−1^ yeast extract and 0.3 mg mL^−1^ cysteine-HCl. Ampicillin (100 μg mL^−1^) was added when appropriate.

### DNA manipulation

Standard DNA recombinant procedures, such as DNA isolation, restriction endonuclease digestion, ligation, the transformation of competent *E. coli* cells, and agarose gel electrophoresis, were performed as described by Sambrook *et al.* (37). Chromosomal DNA was isolated from *P. pantotrophus* GB17 cells using the Dr. GenTLE (from yeast) High Recovery DNA extraction kit (Takara Bio, Otsu, Japan).

### DNA amplification

We used Tks Gflex DNA polymerase (Takara Bio) to improve the fidelity of the PCR assay for *soxX*, *soxY*, *soxZ*, *soxA*, *soxB*, and *soxCD* genes. The reaction mixture (50 μL in total) contained 25 μL of 2× Gflex PCR buffer (containing 2 mM of Mg^2+^ and 400 μM dNTPs; Takara Bio), 1 μL of Tks Gflex DNA polymerase (Takara Bio), 0.01 nM of each primer, and 1 μL of a DNA template, and the volume was adjusted with nuclease-free water (Roche Diagnostics, Indianapolis, IN, USA). The reaction was performed for 30 cycles under the following conditions: initial denaturation at 94°C for 1 min, denaturation at 98°C for 10 s, annealing at 48°C for 15 s, and extension at 68°C for 2.5 min.

### Cloning of sox genes from *P. pantotrophus* GB17

Six *sox* genes (*soxX*, *soxY*, *soxZ*, *soxA*, *soxB*, and *soxCD*) were amplified by PCR from the 5′ terminus of the *sox* gene (13 kbp): the *soxX* fragment (474 bp), *soxY* fragment (423 bp), *soxZ* fragment (330 bp), *soxA* fragment (873 bp), *soxB* fragment (1695 bp), and *soxCD* fragment (2431 bp). These short double-stranded DNAs were compatible with a *Bam*HI site on the 5′-end and a *Hind*III site on the 3′-end. Primers were designed to create *Bam*HI and *Hind*III restriction sites (underlined) within the PCR product (Table 1). The pQE30 vector (Qiagen, Tokyo, Japan) was used for the construction of histidine-tagged recombinant proteins. Each DNA fragment, containing a *sox* gene, was digested with *Bam*HI and *Hind*III and ligated into the pQE30 vector for the expression of 6×His-tagged fusion proteins. In order to obtain the Sox products of *P. pantotrophus* GB17, *E. coli* XL II competent cells were transformed with each resulting plasmid (*soxX*, *soxY*, *soxZ*, *soxA*, *soxB*, and *soxCD*). Positive colonies were selected and re-plated on tryptic soy (TS; Becton, Dickinson, and Company) agar containing ampicillin (100 μg mL^−1^). The nucleotide sequences of the inserted fragments were confirmed by PCR in order to verify that the fragments were correct and did not contain nucleotide substitutions or deletions. A BLAST nucleotide sequence analysis was also performed for DNA sequence identification.

### Preparation of recombinant Sox proteins

Transformants were grown in LB medium with ampicillin (100 μg mL^−1^) at 37°C until optical density at 550 nm (OD_550_) reached 0.5. Isopropyl-β-D(–)-thiogalactopyranoside was added to the culture at a final concentration of 1 mM, and cultures were grown for an additional 4 h. Cells were harvested by centrifugation (5,000×*g*, 4°C, 15 min) and lysed using an ultrasonic sonifier equipped with a microtip (Model W-220-F; Heat Systems Ultrasonics, Plainview, NY, USA) on ice. The cell extract was obtained by centrifugation (10,000×*g*, 4°C, 15 min) and subjected to Ni-NTA resin (Qiagen) affinity column chromatography. Purification procedures followed the manufacturer’s instructions, and the purities of recombinant Sox proteins were analyzed using SDS-PAGE. Eluted proteins were refolded by sequential dialysis against a urea-decreasing phosphate buffer at 4°C (48). The amounts of proteins were assessed using the Lowry method with bovine serum albumin as the standard (19).

### SDS-PAGE and Western blotting

SDS-PAGE was performed using 15% polyacrylamide gels according to the method of Laemmli (17). After electrophoresis, the gel was stained with Coomassie brilliant blue R-250. The low molecular weight electrophoresis calibration kit (Amersham Pharmacia Biotech, Uppsala, Sweden) was used for molecular mass markers. In Western blotting, proteins subjected to SDS-PAGE were transferred electrophoretically to a nitrocellulose membrane according to the method of Burnette (8). After blocking with 2% skimmed milk in Tris-buffered saline (20 mM Tris, 150 mM NaCl, pH 7.2) containing 0.1% Triton X-100 (TBS-T), the membrane was reacted with a HRP-conjugated mouse anti-6×His monoclonal antibody (Wako Pure Chemical, Osaka, Japan). The membrane was washed three times with TBS-T, and fluorescence detection was performed using a chemiluminescence detection system (Clarity Western ECL blotting, Bio-Rad Laboratories, Hercules, CA, USA).

### Enzyme assay

We investigated the ability of a rSox enzyme mixture reconstituted from rSoxX, rSoxY, rSoxZ, rSoxA, rSoxB, and rSoxCD to degrade H_2_S generated from sodium hydrogen sulfide (NaHS). The assay mixture (1 mL) for the assessment of H_2_S-degrading activity contained various amounts of *P. pantotrophus* GB17 cells or rSox enzymes and 20 nmol of NaHS to generate H_2_S in 10 mM phosphate buffer (pH 7.0). All reactions were performed in sterile 15-mL polypropylene tubes sealed with a silicone plug. NaHS was added to start the reaction. After an incubation at 37°C for 2 h, a sample (2.5 mL) of the vapor above the assay mixture in the tube was removed using a gas-tight syringe and analyzed by gas chromatography (model GC-2014; Shimadzu, Kyoto, Japan) using a glass column packed with 25% β,β′-oxydipropionitrile on a 60–80-mesh Chromosorb W AW-DMCS-ST device (Shimadzu) fit with a flame photometric detector at 70°C. The concentration of VSCs was measured using standard H_2_S and CH_3_SH gas prepared with a Permeater PD-1B (Gastec, Ayase, Japan).

The assay mixture (1 mL) used to assess rSox enzyme activity to degrade VSCs produced by periodontopathic bacteria was prepared following the method of Yoshimura *et al.* (49). Briefly, bacterial strains were grown at 37°C until an OD_550_ of approximately 0.6 was reached. Cells were harvested and washed three times with buffered salt solution (40 mM potassium phosphate buffer, 50 mM NaCl, pH 7.7). Cells were suspended in buffered salt solution to an OD_550_ of 0.3 for *F. nucleatum* ATCC10953 and OD_550_ of 1.0 for *P. gingivalis* W83. In order to measure H_2_S, a reaction mixture was prepared consisting of 100 μL of a *F. nucleatum* ATCC10953 or *P. gingivalis* W83 cell suspension and 0.5 nmol each of rSoxX, rSoxY, rSoxZ, rSoxA, rSoxB, and rSoxCD. Buffered salt solution was then added to the tube, which was sealed with a silicone plug. The reaction was initiated by adding 30 μL of 33 mM L-cysteine. In order to measure CH_3_SH, 30 μL of 33 mM L-methionine was added instead of L-cysteine. Assay mixtures were maintained at 37°C and, after a 2-h incubation, the reactions were stopped by adding 500 μL of 3 M phosphoric acid. A sample (2.5 mL) of the vapor above the assay mixture was analyzed by gas chromatography 10 min later, as described above.

The assay mixture used to assess the degrading activity of H_2_S produced by oral bacteria was prepared as described by Tonzetich and Johnson (44) with modifications. Paraffin-stimulated whole saliva was collected from three healthy and non-smoking participants (28–58 years of age), which was approved by the Ethics Committee of Kagoshima University (authorization number 572). The bacterial sediment was collected by centrifugation (10,000×*g*, 4°C, 15 min) and washed three times with buffered salt solution. The bacterial sediment was disrupted by ultrasonication on ice and suspended in buffered salt solution to an OD_550_ of 1.0 in order to measure extracellular H_2_S produced from L-cysteine by oral bacteria. The reaction mixture (3 mL) consisted of a 2-mL bacterial suspension and 0.5 nmol each of rSoxX, rSoxY, rSoxZ, rSoxA, rSoxB, and rSoxCD in buffered salt solution. Subsequent procedures were as described above.

In order to evaluate heat stability, rSox enzymes were mixed and heated at 25 to 100°C for 30 min and then subjected to the H_2_S-degrading assay. The effects of pH on rSox enzyme activity were examined using 10 mM phosphate buffer with pH 4 to 9.

### Statistical analysis

Data were averaged for three independent experiments. Statistical analyses were performed using the Student’s *t*-test or a one-way analysis of variance (ANOVA) followed by Dunnett’s test. *P*-values<0.05 were considered to indicate significance.

## Results

### Purification of recombinant Sox proteins

**Fig. 1:**
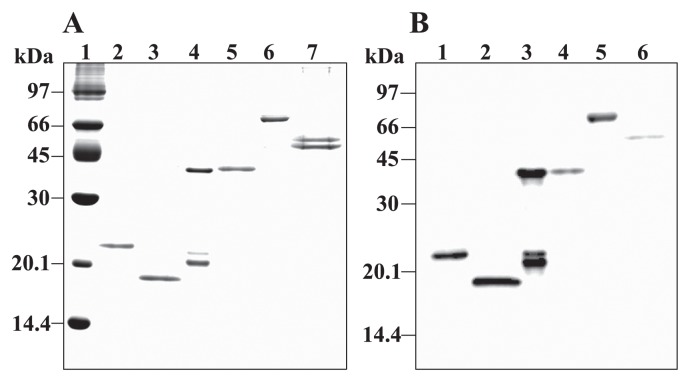
SDS-PAGE (A) and Western blotting (B) analyses of purified rSox proteins. (A) rSox proteins were suspended in SDS-PAGE reducing buffer (1% SDS, 1% 2-mercaptoethanol) and heated at 100°C for 3 min. Samples were subjected to SDS-PAGE (15% polyacrylamide), and the gel was stained with Coomassie brilliant blue R-250. Lanes: 1, molecular mass markers; 2, rSoxX (3 μg); 3, rSoxY (3 μg); 4, rSoxZ (3 μg); 5, rSoxA (3 μg); 6, rSoxB (3 μg); 7, rSoxCD (6 μg). (B) rSox proteins on the gel were transferred electrophoretically to a nitrocellulose membrane, and the membrane was reacted with an antibody against 6×His. Lanes: 1, rSoxX (1.5 μg); 2, rSoxY (1.5 μg); 3, rSoxZ (1.5 μg); 4, rSoxA (1.5 μg); 5, rSoxB (1.5 μg); 6, rSoxCD (3 μg).

Fig. 1A shows purified recombinant proteins. rSoxX migrated at 23 kDa; rSoxY at 18 kDa; rSoxZ at 21, 22, and 43 kDa; rSoxA at 43 kDa; and rSoxB at 68 kDa (Fig. 1A). In the Western blot analysis, the anti-6×His antibody reacted with the rSoxX, rSoxY, rSoxZ, rSoxA, and rSoxB proteins (Fig. 1B). rSoxCD was separated into two bands (51 and 54 kDa) by SDS-PAGE, and the 54-kDa band reacted with the anti-6×His antibody. Since 6×His was attached to rSoxC, the 51-kDa band appeared to be rSoxD and the 54-kDa band rSoxC. rSoxZ showed three bands and the antibody to 6×His reacted with all of these components; therefore, the 43-kDa band appeared to be a dimer, and the 21-kDa band lacked some portion of rSoxZ (the 22-kDa band).

### *P. pantotrophus* GB17 activity to degrade H_2_S

**Fig. 2:**
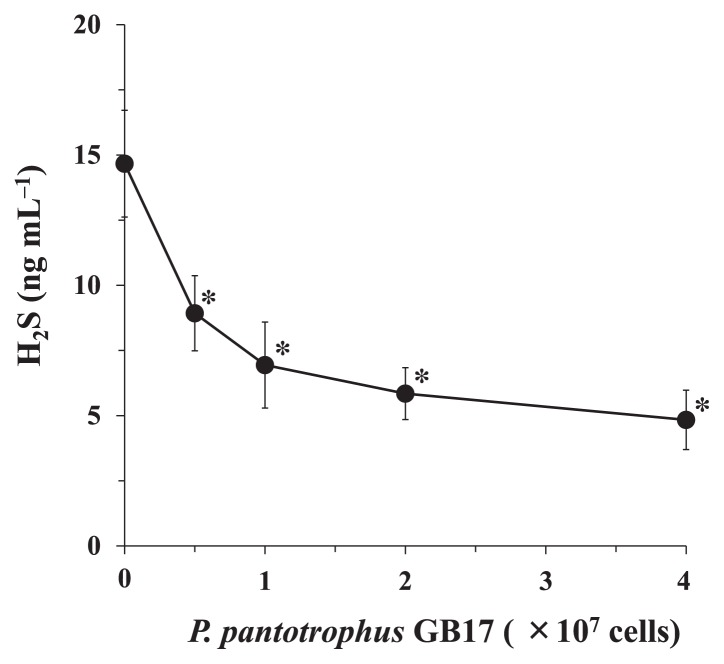
*P. pantotrophus* GB17 activity to degrade H_2_S generated from NaHS. Various amounts of a cell suspension of *P. pantotrophus* GB17 were incubated with 20 nmol NaHS at 37°C for 2 h. Values are the means±SDs of three independent experiments. *P<0.05 significantly different from the control (no *P. pantotrophus* GB17 cells), as assessed by ANOVA followed by Dunnett’s test.

Whole cells of *P. pantotrophus* GB17 were examined for the degradation of H_2_S generated from NaHS. *P. pantotrophus* GB17 cells showed H_2_S-degrading activity, and H_2_S concentrations decreased in a cell number-dependent manner (Fig. 2). Approximately 50% of H_2_S was degraded when a bacterial suspension containing 1.0×10^7^ cells was used.

### rSox enzyme activity to degrade H_2_S

**Fig. 3:**
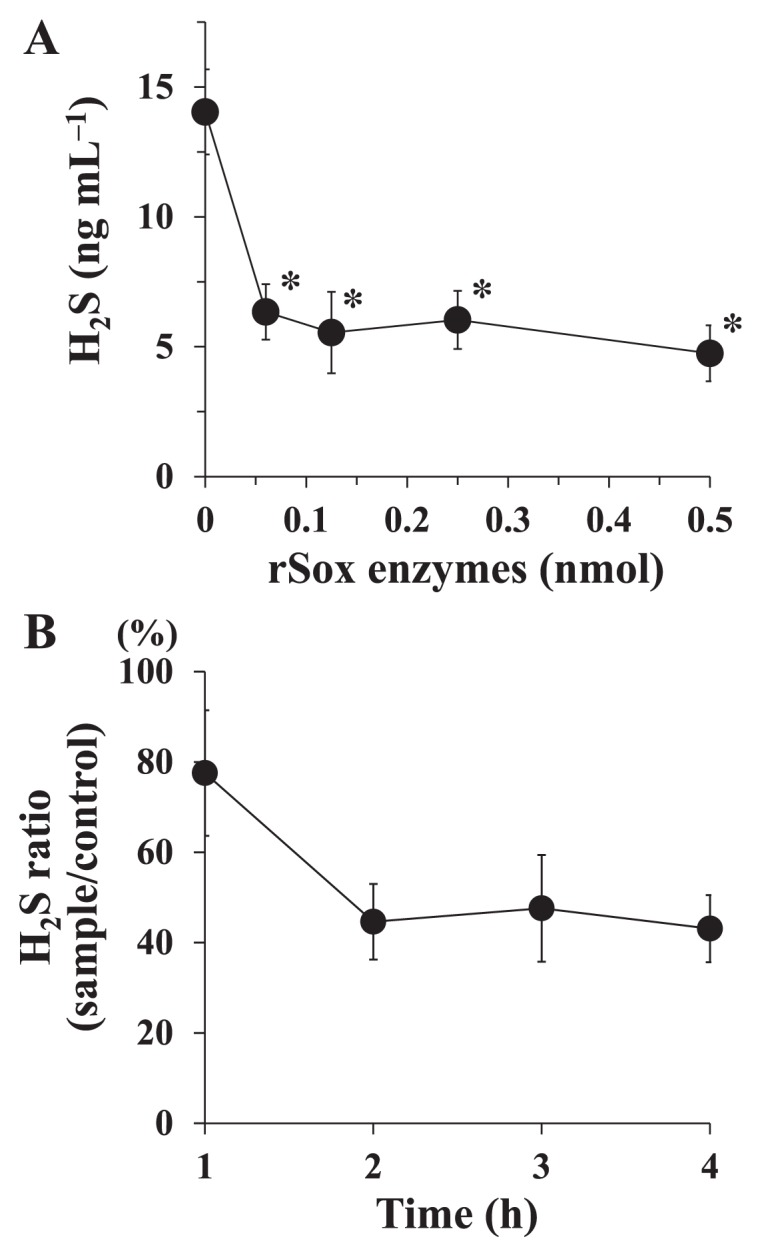
rSox enzyme activity to degrade H_2_S generated from NaHS. (A) Various amounts of the rSox enzyme mixture were reacted with 20 nmol NaHS at 37°C for 2 h. Values are the means±SDs of three independent experiments. *P<0.05 significantly different from the control (no rSox enzymes), as assessed by ANOVA followed by Dunnett’s test. (B) The rSox enzyme mixture (0.125 nmol each enzyme) was reacted with 20 nmol NaHS at 37°C for 1 to 4 h. The assay was performed with (sample) or without (control) the rSox enzyme mixture. Values are the means±SDs of three independent experiments.

**Fig. 4:**
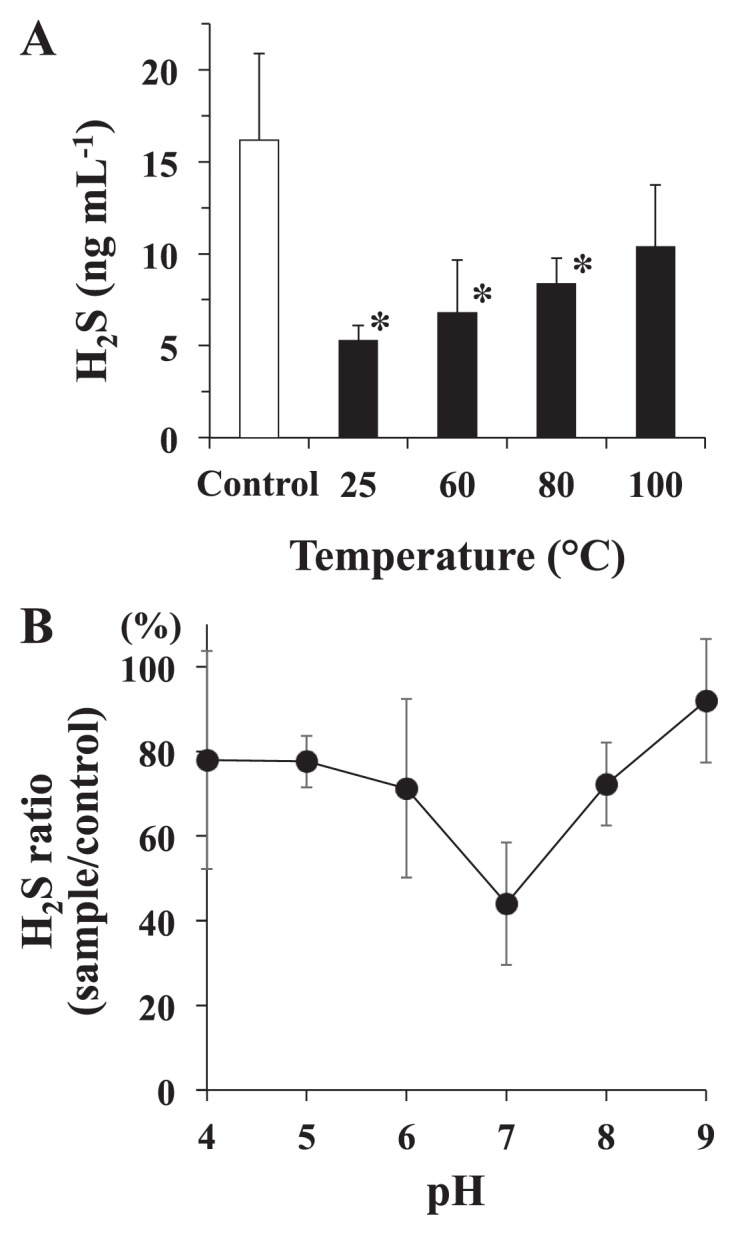
Heat stabilities of rSox enzymes (A) and effects of pH on H_2_S-degrading activities of rSox enzymes (B). (A) After the rSox enzyme mixture (0.125 nmol of each enzyme) was treated at 25 to 100°C for 30 min, samples were reacted with 20 nmol NaHS at 37°C for 2 h. Open bar, control (no rSox enzymes); solid bars, rSox enzymes. Values are the means±SDs of three independent experiments. *P<0.05 significantly different from the control, as assessed by ANOVA followed by Dunnett’s test. (B) The assay was performed with (sample) or without (control) the rSox enzyme mixture (0.125 nmol each enzyme) in 10 mM phosphate buffer (pH 4 to 9) containing 20 nmol NaHS at 37°C for 2 h. Values are the means±SDs of three independent experiments.

A combination of the six rSox proteins (rSoxX, rSoxY, rSoxZ, rSoxA, rSoxB, and rSoxCD) was investigated for its ability to degrade H_2_S generated from NaHS. After being incubated, the rSox enzyme mixture degraded H_2_S in a dose-dependent manner (Fig. 3A). The concentration of H_2_S significantly decreased and reached a stable level after 0.125 nmol of proteins was used. In time-course experiments, the ratio of the H_2_S concentration (sample/control) decreased to approximately 45% in the 2-h incubation and was stable thereafter (Fig. 3B). In the thermal stability study, the amount of H_2_S in the assay mixture increased gradually as the temperature was raised (Fig. 4A). However, the amount of H_2_S was still significantly lower than that with the control, even after heating at 80°C. After heating at 100°C, no significant difference was observed in the amount of H_2_S from that with the control. Regarding the effects of pH on rSox enzyme activity, the percentage of the H_2_S concentration (sample/control) was the lowest at pH 7.0, and higher values were observed at other pH values (Fig. 4B).

### Effects of each component of the rSox enzyme mixture on H_2_S degradation

Table 2 shows the effects of the components of the rSox enzyme mixture on the degradation of H_2_S. In this experiment, the rSoxX and rSoxA or rSoxY and rSoxZ pair was omitted from the assay because these pairs of enzymes work together in the Sox system (12, 34). When a mixture of rSox enzymes reconstituted from all rSox enzymes was used, H_2_S levels were significantly lower than those with the control (no rSox enzymes). In other cases where certain rSox enzymes were omitted from the assay, H_2_S levels were not significantly different from the control, and H_2_S ratios relative to control levels were approximately 70%. Bovine serum albumin, which was used as a non-sulfur-oxidizing control, showed no reducing effect on H_2_S.

### rSox enzyme activity to degrade VSCs produced by periodontopathic bacteria and oral bacteria

We investigated the ability of rSox enzymes to degrade H_2_S produced by *F. nucleatum* ATCC10953 and *P. gingivalis* W83. The rSox enzyme mixture significantly degraded H_2_S produced by the two bacterial strains (Table 3). We also assessed CH_3_SH degradation by the rSox enzyme mixture and found that it only slightly decreased CH_3_SH levels from those with the control (no enzymes). Oral bacteria were collected from human saliva and used to produce H_2_S. The rSox enzyme mixture significantly degraded H_2_S produced from the oral bacteria of all subjects; however, the degree of degradation differed among subjects (Table 4).

## Discussion

In the present study, we demonstrated the degradation of H_2_S by rSox enzymes from *P. pantotrophus* GB17 in view of oral malodor prevention. We produced rSox enzymes to examine their VSC-degrading activities. We attempted to construct rSoxXA, rSoxYZ, rSoxB, and rSoxCD proteins; however, only rSoxB and rSoxCD proteins were successfully obtained. Thus, we constructed each enzyme as rSoxX, rSoxY, rSoxZ, and rSoxA. We demonstrated that the combination of rSoxX, rSoxY, rSoxZ, rSoxA, rSoxB, and rSoxCD proteins degraded H_2_S, and this activity was almost constant after reaching the maximum effect (Fig. 3A). Regarding the use of recombinant Sox enzymes, rSoxXA expressed in *E. coli* was found to be functional in the reconstituted enzyme system to activate H_2_S-dependent cytochrome *c* reduction (35). This study is the first to describe a Sox system composed of each recombinant Sox protein for degrading H_2_S.

High cytochrome *c*-reducing activity by the Sox system was observed using H_2_S as the substrate (34), and SoxXA, SoxYZ, SoxB, and SoxCD were required to achieve optimal activity (12, 34). In the present study, we demonstrated that all of the enzymes—rSoxX, rSoxY, rSoxZ, rSoxA, rSoxB, and rSoxCD—were necessary for H_2_S degradation (Table 2). The central protein of the Sox enzyme system is SoxYZ, to which the sulfur substrate is covalently linked, oxidized, and finally released as sulfate by three other Sox proteins (11). SoxXA binds the sulfur substrate and covalently links it to the thiol of the single cysteine 110 residue of the SoxY subunit. SoxY forms a complex with SoxZ. The outer sulfur atom is oxidized by sulfur dehydrogenase SoxCD, yielding SoxY-cysteine-S-sulfate. Finally, SoxY-cysteine-S-sulfate is hydrolyzed by the dimanganese SoxB protein to yield sulfate and regenerate SoxY for a new reaction cycle of SoxYZ (13, 36). When SoxB or SoxYZ was omitted from the assay mixture, residual H_2_S-dependent activities decreased to approximately 12 or 9%, respectively, that of the mixture of SoxXA, SoxYZ, SoxB, and SoxCD (34). SoxCD was also required to achieve the maximum rate of H_2_S-dependent cytochrome *c* reduction, showing a residual rate of 19% that of the mixture of all Sox enzymes when SoxCD was omitted. The results obtained in this study are consistent with these findings.

In *P. pantotrophus* GB17, each *sox* gene encodes a protein with a different molecular mass: SoxX, 16,421 Da; SoxY, 13,830 Da; SoxZ, 11,850 Da; SoxA, 31,884 Da; SoxB, 61,897 Da; SoxC, 43,442 Da; and SoxD, 37,637 Da (12). In the present study, the estimated molecular masses of rSox enzymes by SDS-PAGE were larger than those predicted by the nucleotide sequences (Fig. 1A). All rSox proteins, except for rSoxD, had a 6×His tag; however, the differences observed were larger than the size of 6×His (approximately 1.2 kDa). Previous studies (12, 29) also showed that purified Sox proteins had different molecular masses from the predicted ones (SoxX, 16 kDa; SoxY, 12 kDa; SoxZ, 16 kDa; SoxA, 29 kDa; SoxB, 59 kDa; SoxC, 47 kDa; and SoxD, 50 kDa) under denaturing SDS-PAGE. Friedrich *et al.* (12) reported that the molecular mass of SoxY assessed by SDS-PAGE differed from that predicted by the nucleotide sequence due to an unknown reason. Furthermore, Quentmeier *et al.* (29) found that the SoxC and SoxD proteins had different molecular masses assessed by SDS-PAGE from those deduced from the nucleotide sequence of the mature protein. Mobility on SDS-PAGE gels may be affected by the amino acid composition, glycosylation, or phosphorylation of the protein. In the present study, amino acid compositions and predicted pI values were obtained using the Compute pI/Mw tool in ExPASy. None of the rSox enzymes appeared to have a unique amino acid composition, and pI values were between 4.5 and 6.6. These characteristics did not appear to affect the mobilities of the proteins on SDS-PAGE gels. A BLAST nucleotide sequence analysis showed the identities of all of the *sox* genes of *P. pantotrophus* GB17 used in this study; therefore, the reason for the differences observed in mass remains unclear.

The optimal pH for the H_2_S-degrading activities of rSox enzymes was 7.0, and activities were stable at 25–80°C. *P. pantotrophus* was originally isolated as *Thiosphaera pantotropha* (33), was reclassified as *P. denitrificans* (20), and then renamed (31). *T. pantotropha* GB17 obtains energy through the Sox system and grows at pH 6.5–10.5, with an optimum pH of 8.0, and at a temperature of 15–42°C, with an optimum at 37°C (33). Another study demonstrated the optimal growth conditions of *P. denitrificans* at pH 7.5–8.0 and 30–37°C (11). These findings suggest that the Sox system functions under these pH and temperature conditions, which appears to be reasonable. We also found that whole cells from *P. pantotrophus* GB17 degraded H_2_S, even after heating at 100°C (data not shown). Thermophilic sulfur-oxidizing bacteria have recently been isolated from geothermal fields (25, 39). Although it currently remains unclear whether these bacteria contain the Sox system, Sox enzymes may possess thermophilic properties. Ghosh *et al.* (14) suggested that the Sox system originated in ancient thermophilic bacteria and evolved through extensive horizontal gene transfer. Further studies are needed in order to clarify the mechanisms responsible for the thermal stabilities of rSox enzymes. The heat stabilities of rSox enzymes may be advantageous in potential clinical applications, because intra-oral temperature changes after the ingestion of various cold/hot meals and beverages (4, 22).

In the present study, we demonstrated that rSox enzymes significantly degraded H_2_S produced by periodontopathic bacteria. The reducing effects of rSox enzymes in reaction mixtures with *P. gingivalis* or *F. nucleatum* were weaker than those obtained in reaction mixtures with NaHS. We examined the effects of rSox enzymes on the growth of these bacterial cells and found no significant effects (data not shown). rSox enzymes also showed the potential to decrease CH_3_SH produced by *P. gingivalis*. The Sox system, reconstituted from SoxXA, SoxYZ, SoxB, and SoxCD, mediates thiosulfate-, sulfite-, sulfur-, and H_2_S-dependent cytochrome *c* reduction (12, 34); however, CH_3_SH-dependent reduction has not yet been demonstrated. The availability of CH_3_SH as a substrate for the Sox system needs to be clarified in further studies. We also showed that H_2_S produced by oral bacteria collected from human saliva decreased after an incubation with rSox enzymes. These results indicate the potential of rSox enzymes to reduce H_2_S produced in the oral cavity.

Many randomized controlled trials have reported the reducing effects of mouth rinses containing chlorhexidine, cetylpyridinium chloride, or metal ions on oral malodor, whereas several studies have reported no significant effects (7, 30, 40). Systematic reviews showed that available data are insufficient to assess precision (5) and concluded that the strength of the recommendation to use these ingredients for oral malodor reduction is graded as ‘weak’ (40). It currently remains unclear whether Sox enzyme-reducing effects on H_2_S are superior to existing components. Chlorhexidine and cetylpyridinium chloride act to suppress bacterial growth and may be accompanied by several adverse effects in oral tissues. The Sox system appears to be advantageous in this point because its target is H_2_S produced by oral bacteria, not oral bacteria or oral tissues. Furthermore, previous studies reported that H_2_S participates in physiological regulation in the human body (28, 46, 50). Therefore, the Sox system has potential as an effective agent for controlling body systems as a H_2_S regulator.

More data are needed for the final goal of clinical applications of this enzyme system. The toxicity of the Sox enzyme system as well as the stabilities of rSox enzymes against proteases present in saliva need to be examined. The maximum effect of the rSox enzyme mixture appeared 2 h after the reaction started (Fig. 3B). In the present study, we examined rSox enzyme activities using assay mixtures in a tube sealed with a silicon plug. Since many components including the type and amount of the sulfur source, the pH of oral fluids, saliva flow rates, and oxygen levels in saliva and dental plaque affect the formation of VSCs in the mouth (23), the duration needed to achieve the maximum effect of the rSox enzyme mixture in the oral cavity currently remains unclear. We expect the reducing effects of rSox enzymes to last for a long time. Although their effects lasted for 4 h in the assay used in the present study, the duration of these effects in the oral environment has yet to be established. Further studies are needed in order to clarify these issues before clinical applications are realized.

In conclusion, we demonstrated that the combination of seven rSox enzymes from *P. pantotrophus* GB17 exhibited H_2_S-reducing activity. These results suggest the potential of the rSox system to prevent oral malodor.

## Figures and Tables

**Table 1:** Oligonucleotide primers used in this study

Fragment	Primer	Sequence (5′ to 3′)^a^
rSoxX	rSoxX-Forward	AGGGATCCATGAGCAGCCATCTATGG
	rSoxX-Reverse	CTAAGCTTGTCGAGCCTGTAGAGATC
rSoxY	rSoxY-Forward	AGGGATCCATGATCCTTTCAAGACGC
	rSoxY-Reverse	GCAAGCTTAATCTCCTGTTACTGGAC
rSoxZ	rSoxZ-Forward	AGGGATCCATGGCAGATGATGCAAAG
	rSoxZ-Reverse	TGAAGCTTGATGTTGCGGCGCTTAGG
rSoxA	rSoxA-Forward	GAGGATCCATGCCGCGCTTTACCAAG
	rSoxA-Reverse	GGAAGCTTGAAGCATTGCCCTTTCGA
rSoxB	rSoxB-Forward	ACGGATCCATGATTACCCGACGTGAG
	rSoxB-Reverse	CAAAGCTTAACGCTCCTTCGTGATTG
rSoxCD	rSoxCD-Forward	GTGGATCCATGAAAGACGAGCTCACC
	rSoxCD-Reverse	AGAAGCTTCTGTCTCATGCGTCACTT

a Nucleotides underlined in each primer sequence show the position of the restriction endonuclease site incorporated to facilitate cloning.

**Table 2:** Effects of each rSox enzyme on the degradation of H_2_S generated from NaHS

Sox enzymes added to the assay							H_2_S (ng mL^−1^)^a^	Ratio to the control (%)

X	A	Y	Z	B	CD	BSA^b^		
−	−	−	−	−	−	−	16.9±3.8	
+	+	+	+	+	+	−	6.4±1.1*	37.9
−	−	+	+	+	+	−	11.9±2.5	70.4
+	+	−	−	+	+	−	11.5±2.1	68.0
+	+	+	+	−	+	−	12.0±4.1	71.0
+	+	+	+	+	−	−	12.0±1.5	71.0
−	−	−	−	−	−	+	17.2±4.7	101.8

In order to assess the role of each rSox component, an enzyme mixture was prepared as shown in the table. The rSox enzyme mixture (0.125 nmol each) prepared was reacted with 20 nmol of NaHS at 37°C for 2 h.

a Values are the means±SDs of three independent experiments.

b BSA, bovine serum albumin (0.125 nmol) was used as a non-sulfur-oxidizing control.

* *P*<0.05 significantly different from the control (without rSox enzymes or BSA), as assessed by ANOVA followed by Dunnett’s test.

**Table 3:** rSox enzyme activity to degrade VSCs produced by periodontopathic bacteria

Bacterial strain	H_2_S (ng mL^−1^)^a^		CH_3_SH (ng mL^−1^)^a^	
	
	rSox enzymes		rSox enzymes	
	
	(−)	(+)	(−)	(+)
*F. nucleatum* ATCC10953	26.7±2.2	12.1±1.9*	ND	ND
*P. gingivalis* W83	19.6±4.5	9.1±3.5*	22.0±2.9	15.9±1.8

a Values are the means±SDs of three independent experiments.

* *P*<0.05 significantly different from the control (without rSox enzymes), as assessed by the Student’s *t*-test.

ND, not determined.

**Table 4:** rSox enzyme activity to degrade H_2_S produced by oral bacteria

Subject	H_2_S (ng mL^−1^)^a^		Ratio (%)(rSox/Control)

	Control	rSox enzymes	
A	23.8±3.8	13.3±4.7*	55.9
B	22.3±6.3	6.5±0.7*	29.1
C	8.1±1.1	2.7±0.2*	33.3

a Values are the means±SDs of three independent experiments.

* *P*<0.05 significantly different from the control (without rSox enzymes), as assessed by the Student’s *t*-test.
